# FOXC1: A Key Transcription Factor of VSMC‐Derived Foam Cell Formation in Atherosclerotic Plaque Instability

**DOI:** 10.1002/kjm2.70269

**Published:** 2026-07-20

**Authors:** Ling‐Lin Qian, Xue‐Ling Li, Li‐Hong Wang, Wei Yang, Yu Jiang

**Affiliations:** ^1^ Heart Center, Department of Cardiovascular Medicine Zhejiang Provincial People's Hospital (Affiliated People's Hospital), Hangzhou Medical College Zhejiang Hangzhou China; ^2^ Center for General Practice Medicine, General Practice and Health Management Center Zhejiang Provincial People's Hospital (Affiliated People's Hospital), Hangzhou Medical College Zhejiang Hangzhou China

**Keywords:** atherosclerosis, bioinformatics analysis, foam cell, plaque instability, vascular smooth muscle cell

## Abstract

The instability of atherosclerotic plaques, particularly intraplaque hemorrhage (IPH), drives life‐threatening cardiovascular events, a process in which vascular smooth muscle cell (VSMC)‐derived foam cells play a significant role. We aim to identify key biomarkers associated with VSMC‐derived foam cells and IPH by analyzing data from human IPH datasets and VSMC‐derived foam cell datasets (GSE163154, GSE68021, GSE28829, and GSE43292). Weighted gene co‐expression network analysis (WGCNA), differential expression analysis, and machine learning algorithms (LASSO and SVM‐RFE) were employed to identify hub genes. The identified genes were validated in independent datasets and in experimental models, including oxidized low‐density lipoprotein‐stimulated VSMCs and atherosclerotic aortic tissues of high‐fat diet‐fed ApoE^−^/^−^ mice. Transcription factor (TF) prediction and dual‐luciferase reporter assays were performed to explore upstream regulatory mechanisms. We identified CD68 and CYBA as key hub genes significantly upregulated in both VSMC‐derived foam cells and unstable atherosclerotic plaques, with high diagnostic accuracy (AUC > 0.9) by ROC analysis. Experimental validation confirmed their upregulated expression. FOXC1 was identified as a common upstream transcription factor regulating both CD68 and CYBA. FOXC1 protein levels were elevated in VSMC‐derived foam cells, and dual‐luciferase assays confirmed its direct activation of CD68 and CYBA promoters. FOXC1 overexpression promoted CD68 and CYBA expression and enhanced lipid accumulation in VSMCs, while FOXC1 knockdown exerted opposite effects. Immune infiltration analysis revealed significant correlations between these hub genes and immune cell populations in unstable plaques. This study identifies FOXC1 as a key TF regulating CD68 and CYBA expression, thereby promoting VSMC‐derived foam cell formation and plaque instability.

## 1 Introduction

Atherosclerosis (AS) is a chronic inflammatory disorder pathologically characterized by the progressive accumulation of plaque within the arterial walls [1, 2]. The development of atherosclerotic plaques encompasses a series of interconnected processes, including endothelial dysfunction, oxidized low‐density lipoprotein (ox‐LDL) deposition, inflammatory responses, foam cell generation, vascular smooth muscle cell (VSMC) migration and fibrous cap formation [3]. Among these pathophysiological processes, plaque instability emerges as the most significant clinical hazard, acting as the principal precipitating factor for life‐threatening conditions such as ischemic stroke and acute coronary syndrome [4]. A critical determinant of plaque vulnerability is the excessive aggregation of lipid‐laden foam cells within the atherosclerotic lesions. These foam cells originate from the uncontrolled uptake of ox‐LDL via scavenger receptors (SRs), which is a hallmark feature of unstable plaques [5]. Traditionally, macrophages have been regarded as the primary source of foam cells. However, recent studies have unveiled that VSMC‐derived foam cells make a substantial contribution to the overall foam cell population [6, 7], indicating that VSMC are also a considerable source of foam cells.

Within the subendothelial region, ox‐LDL is internalized by VSMCs through SRs, including lectin‐like oxidized low‐density lipoprotein receptor‐1, scavenger receptor class A, cluster of differentiation 36 and C‐X‐C motif chemokine ligand 16/scavenger receptor for phosphatidylserine and oxidized lipoprotein. This internalization process drives the transdifferentiation of VSMCs into foam cells [8]. Upon stimulation with ox‐LDL, VSMCs experience phenotypic conversion, which is manifested by downregulation of the α‐smooth muscle actin (a VSMC marker) and the acquisition of the cluster of differentiation 68 (CD68) (a macrophage marker) [9]. Despite these documented observations, the specific molecular mechanisms that underpin the transformation of VSMCs into foam cells remain elusive and necessitate further in‐depth investigation.

The rapid progress in microarray technology and bioinformatics tools has facilitated high‐throughput screening and precise identification of molecular targets in biomedical research, thereby providing fresh perspectives on the pathogenesis of AS [10, 11]. This study is designed with the objective of identifying novel molecular targets specific to VSMC‐derived foam cells within unstable atherosclerotic plaques. Notably, within the context of this study, the intraplaque hemorrhage (IPH) group was defined as representing unstable plaques. Four independent microarray datasets were enrolled for research: human carotid atheroma IPH tissues and non‐IPH tissues (GSE163154, GSE28829 and GSE43292); ox‐LDL‐treated human VSMC (hVSMC) and control hVSMC (GSE68021).

This study was conducted based on a multi‐step strategy (Figure S1). In brief, the overlapping differentially expressed genes (ODEGs) between hVSMC‐derived foam cells and human carotid atheroma IPH tissues in the two datasets (GSE68021 and GSE163154) were screened out. We applied Gene Ontology (GO) and Kyoto Encyclopedia of Genes and Genomes (KEGG) enrichment to scientifically analyze the functions of ODEGs. The CytoHubba plugin based on protein–protein‐interaction (PPI) network combined with the machine learning algorithm was then used to pick out the hub genes of ODEGs. These hub genes were further validated in other datasets (GSE28829 and GSE43292), as well as the models of mouse VSMC‐derived foam cell and atherosclerotic mouse. By employing a comprehensive bioinformatics strategy, we successfully delineated the molecular signatures and immune characteristics of genes associated with VSMC‐derived foam cells in the context of IPH. Finally, the upstream transcription factor (TF) that governs hub gene expression was identified. This integrated strategy provides a framework for understanding VSMC‐derived foam cell formation and identifying potential therapeutic targets for unstable AS.

## 2 Materials and Methods

### 2.1 Data Source

In the present study, we retrieved four gene expression profiles (GSE163154, GSE68021, GSE28829 and GSE43292) from the gene expression omnibus (GEO) database (https://www.ncbi.nlm.nih.gov/gds/?term=). The GSE163154 dataset consisted of carotid artery plaque samples obtained from patients. These patients were classified into two groups: the IPH group with 27 samples (*n* = 27) and the non‐IPH group with 16 samples (*n* = 16). The GSE68021 dataset encompassed 12 cases of hVSMC samples, including 9 cases of samples treated with ox‐LDL and 3 cases of control samples. The GSE28829 dataset included 16 cases of advanced atherosclerotic plaques and 13 cases of early‐stage atherosclerotic plaques. The GSE43292 dataset contained 32 cases of carotid atheroma plaques and 32 cases of distant macroscopically intact tissues. For the purposes of our analysis, GSE163154 and GSE68021 were designated as training datasets, whereas GSE28829 and GSE43292 were employed for validation.

### 2.2 Identification of Module Genes Related to AS

In this study, we utilized weighted gene co‐expression network analysis (WGCNA) to pinpoint pivotal gene modules associated with IPH and explore the relationship between gene networks and IPH within the GSE163154 dataset. To initiate the process, we conducted hierarchical clustering analysis to detect any potential outliers. Following this, we opted for a soft threshold of 14 to construct a scale‐free network by employing the “pickSoftThreshold” function. Subsequently, we established the topological overlap matrix and generated the gene dendrogram and module colors based on the dissimilarity degree. Finally, we performed Pearson correlation analysis to ascertain the correlation between gene modules and traits. Through an evaluation of the correlation between modules and clinical characteristics, we identified the module most strongly associated with IPH, which was deemed the key module. The genes within this key module were then subjected to further screening for subsequent analyses.

### 2.3 Identification of Differentially Expressed Genes (DEGs) and ODEGs

We employed the “limma” package in R to perform differential expression analysis on the standardized dataset. The screening criteria were set as *p*‐value < 0.05 and |log_2_FC| > 0.6 to identify DEGs. Following this, we utilized the “ggplot2” package to generate volcano plots, which visually represent the differential expression status of genes by plotting the log_2_FC on the x‐axis and the negative logarithm of the *p*‐value (−log_10_(*p*‐value)) on the *y*‐axis. In addition, we created heatmaps using the “pheatmap” package. Heatmaps offer a comprehensive visualization of gene expression patterns across different samples. The ODEGs were obtained by taking the DEGs of GSE163154 and GSE68021, as well as the genes in the blue module of WGCNA.

### 2.4 Functional Enrichment Analysis

We utilized the online DAVID database (https://david.ncifcrf.gov/) to conduct GO and KEGG enrichment analyses for gene functional annotation. The GO term selects biological processes (BP) and cellular components (CC). We identified significantly enriched terms based on an adjusted *p*‐value < 0.05.

### 2.5 Construction of PPI Network and Preliminary Identification of Hub Genes

To better understand how the ODEGs interact with each other, we used the STRING online website (https://string‐db.org/) to build a PPI network. To make sure the interactions in the network were reliable, we set the minimum interaction score to be more than 0.4. We also got rid of any nodes that weren't connected to others, which made the network clearer. Then, we imported this PPI network into Cytoscape software for further analysis. The hub genes in the PPI network were identified using three topological algorithms in the CytoHubba plugin of Cytoscape: maximum clique centrality (MCC), maximum neighborhood component (MNC), and density of maximum neighborhood component (DMNC). For each algorithm, we picked the top 10 ranked genes and took their intersection.

### 2.6 Optimizing the Identification of Hub Genes by Machine Learning Algorithms

To pinpoint the pivotal hub genes, we adopted two highly reliable machine‐learning methodologies: Least Absolute Shrinkage and Selection Operator (LASSO) and Support Vector Machine‐Recursive Feature Elimination (SVM‐RFE). LASSO was performed using the R package “glmnet”, with a binomial response type and an alpha value of 1. The optimal lambda (*λ*) value was determined based on the minimum binomial deviance and the 1‐standard‐error (1‐SE) rule, and visualized via partial likelihood deviance and log(*λ*) curves. SVM‐RFE, a supervised learning algorithm renowned for its high classification accuracy, was implemented with the “e1071” and “MSVM‐RFE” packages. The model incorporated sequential backward elimination to refine feature selection. These genes exhibited the lowest 5‐fold cross‐validation (CV) error and highest 5‐fold CV accuracy, confirming their diagnostic relevance.

### 2.7 Hub Gene Validation in Other Datasets and Receiver Operating Characteristic (ROC) Analysis in GSE163154

We employed the “ggplot2” package in R to generate box plots, aiming to visually depict the expression levels of hub genes between advanced atherosclerotic samples and control samples in the GSE28829 and GSE43292 datasets. Additionally, to evaluate the accuracy of hub genes in the GSE163154 dataset, we conducted ROC curve analysis. This analysis was performed using the “pROC” package. Hub genes with an area under the curve (AUC) > 0.8 were considered valuable for disease diagnosis.

### 2.8 Immune Infiltration Analysis

The relative proportions of immune cell populations in the GSE163154 dataset were quantified via CIBERSORT, a deconvolution algorithm for inferring immune cell composition from bulk gene expression profiles [12]. Subsequently, Spearman's rank correlation analysis was conducted to assess potential associations between the identified immune cell subsets and hub genes.

### 2.9 Prediction of TF Targeting Hub Genes

TFs are a class of DNA‐binding proteins that regulate gene expression at the transcriptional level by binding to specific DNA sequences. To identify potential TFs that may regulate the hub genes identified in this study, we utilized NetworkAnalyst (https://www.networkanalyst.ca/), a publicly accessible online platform for integrative network analysis of gene expression signatures.

### 2.10 Cell Culture

Mouse aortic vascular smooth muscle cells (MOVAS) were purchased from the American Type Culture Collection and cultured in Dulbecco's Modified Eagle Medium (DMEM, HyClone, USA) containing 10% fetal bovine serum (Gibco, USA) and 1% penicillin–streptomycin in an incubator at 37°C with 5% CO_2_. VSMCs were then treated with ox‐LDL (YB‐002, Yiyuan Biotechnology, China) at a concentration of 50 mg/L for 24 h. This treatment was aimed at facilitating lipid phagocytosis and promoting the subsequent formation of VSMC‐derived foam cells. Foam cell formation was assessed through oil red O staining (ORO) and nile red staining (Sigma, St. Louis, USA).

The forkhead box C1 (FOXC1) small interfering RNA (siRNA) and its overexpression plasmid were commercially synthesized (Genepharm, China). They were then transfected into MOVAS separately according to the manufacturer's instructions, followed by intervention with ox‐LDL for 24 h.

### 2.11 Quantitative Real‐Time Polymerase Chain Reaction (qRT‐PCR) of Hub Genes

After extracting total RNA with TRIzol reagent, the mRNA was isolated from MOVAS or the whole aorta, followed by purification using DNase to remove any potential genomic DNA contamination. Subsequently, complementary DNA (cDNA) was synthesized using a Transcriptor First Strand cDNA Synthesis Kit (TaKaRa Bio, Japan). qRT‐PCR was carried out on a StepOnePlus Real‐Time PCR System (Thermo Fisher Scientific). Details regarding the primer sequences are presented in Table S1. The quantified transcript levels from the samples were normalized to the expression level of the β‐actin gene. The data were calculated using the 2^−△△*C*t^ method and are presented as relative expression levels.

### 2.12 Western Blotting

Proteins were extracted from MOVAS under different experimental conditions using RIPA lysis buffer. Protein concentrations were measured via a bicinchoninic acid assay (Beyotime Biotechnology, China). Equal amounts of total protein (20–30 μg) were separated by 10% sodium dodecyl sulfate‐polyacrylamide gel electrophoresis and then transferred onto polyvinylidene fluoride membranes. Following blocking, the membranes were incubated with primary antibodies overnight at 4°C and subsequently with corresponding secondary antibodies. Finally, protein bands were visualized using an enhanced chemiluminescence detection system. The primary antibodies used were against FOXC1 (1:2000; 55365‐1, Proteintech), H4 transcription factor (HINFP) (1:2000; 10066‐2, proteintech), α‐SMA (1:1000; AF1032, Affinity Biosciences), SM22α (1:2000; AF9266, Affinity Biosciences), CYBA (1:2500; DF10099, Affinity Biosciences), CD68 (1:2500; DF7518, Affinity Biosciences), and β‐actin (1:15000; AF7018, Affinity Biosciences).

### 2.13 Luciferase Reporter Assay

A dual‐luciferase reporter assay was conducted to validate the transcriptional regulation of the target gene. The putative promoter region of interest was cloned into a firefly luciferase reporter vector. 293 T cells and MOVAS were separately co‐transfected with the constructed reporter plasmid, an expression plasmid for the TF and a Renilla luciferase plasmid serving as an internal control for normalizing transfection efficiency. After 48 h of transfection, luciferase activities were measured using a dual‐luciferase reporter assay system according to the manufacturer's protocol. Firefly luciferase activity was normalized to Renilla luciferase activity. The experiment was conducted with three independent replicates.

### 2.14 Intracellular Reactive Oxygen Species (ROS) Assay

MOVAS were seeded into a 96‐well plate and treated as indicated (FOXC1 overexpression or knockdown, followed by ox‐LDL stimulation for 24 h). Intracellular ROS levels were detected using a ROS assay kit (Beyotime, China) according to the manufacturer's instructions. In brief, the treated cells were incubated with serum‐free DMEM containing 10 μM DCFH‐DA for 30 min at 37°C in the dark. The cells were then washed three times with serum‐free DMEM, and fluorescence intensity was measured using a fluorescence microplate reader (Thermo Fisher Scientific, USA).

### 2.15 Establishment of an Animal Model of AS

In this study, all mice were purchased from the Changzhou Cavens Laboratory Animal Ltd. (Changzhou, China). Five male ApoE^−/−^ mice and five male C57BL/6 mice, all 8 weeks old, weighed between 18 and 20 g. These mice were fed a high‐fat diet (HFD) containing 10% fat, 52% carbohydrate, 26% protein, 2% cholesterol, and 0.5% sodium cholate for 12 weeks. All procedures were conducted in accordance with the National Institutes of Health Guide for the Care and Use of Laboratory Animals (NIH Publication No. 8523, revised 1996). The mice were housed at room temperature with a 12‐h light/dark cycle and had free access to food and water.

### 2.16 Atherosclerotic Lesion Analysis

After 12 weeks on the HFD, the mice were euthanized by intraperitoneal injection of an overdose of pentobarbital. Subsequently, transcardial perfusion with 10 mL of phosphate‐buffered saline was performed. The heart was embedded in optimal cutting temperature compound, snap‐frozen in liquid nitrogen, and then cross‐sectioned serially at the aortic root level to a thickness of 5 μm. Cryosections of the aortic root were then stained with ORO and counterstained with hematoxylin–eosin. Images were captured using a Leica image analysis system. Quantification was carried out with ImageJ.

### 2.17 Statistical Analysis

All data are presented as the means ± standard deviation, calculated from at least three independent experiments. Intergroup comparisons were conducted using either a two‐tailed Student's *t*‐test (for pairwise comparisons) or one‐way analysis of variance followed by post hoc multiple comparison tests (for comparisons involving more than two groups). All statistical analyses were performed using SPSS software (Version 16.0, Armonk, NY, USA). A *p*‐value < 0.05 was considered statistically significant.

## 3 Results

### 3.1 WGCNA Identifies IPH‐Associated Modules

To systematically identify gene networks associated with unstable atherosclerotic plaques, we performed WGCNA of the GSE163154 dataset. The analysis was conducted using scale‐free topology criteria and mean connectivity measures, with an optimal soft threshold power of 10 being selected to construct the co‐expression network (Figure S2A). Through hierarchical clustering combined with dynamic tree cutting, we identified nine distinct gene co‐expression modules (Figure S2B,C). Module‐trait relationship analysis revealed significant associations with IPH (Figure S2D). Among these modules, the blue module (including 1024 genes) showed the strongest positive correlation with IPH (Pearson's |*r*| > 0.9, *p* < 0.05) and was consequently identified as the most relevant disease‐associated module (Figure S2E).

### 3.2 Identification of DEGs Associated With VSMC‐Derived Foam Cells and IPH

For GSE163154, a total of 1435 DEGs (|log_2_FC| > 0.6 and adjusted *p* < 0.05) were identified from IPH, including 876 upregulated genes and 559 downregulated genes. The DEGs were shown in the volcano plot (Figure S3A). A heatmap displayed the expression levels of the top 50 DEGs in both sample groups (Figure S3B). For GSE68021, 672 genes were found to be upregulated and 578 genes downregulated in VSMC‐derived foam cells and the top 50 statistically significant genes were shown in a heatmap (Figure S3C,D).

### 3.3 Identification of Co‐Expressed DEGs Between VSMC‐Derived Foam Cells and IPH

By intersecting the DEGs of datasets GSE163154 and GSE68021 with the genes from the blue module identified in the WGCNA of GSE163154, we obtained 40 ODEGs (Figure S4A). BP of GO analysis revealed significant enrichment in antigen processing and presentation, zymogen activation, etc. (Figure S4B). For CC terms, the ODEGs were predominantly enriched in secretory granule membrane, lysosomal membrane, etc. Meanwhile, KEGG pathway analysis demonstrated that these ODEGs were prominently enriched in four pathways: phagosome, rheumatoid arthritis, lipid and atherosclerosis, and peroxisome proliferator‐activated receptor signaling pathway (Figure S4C).

### 3.4 Hub Gene Screening via PPI Network Analysis and Machine Learning

**FIGURE 1:**
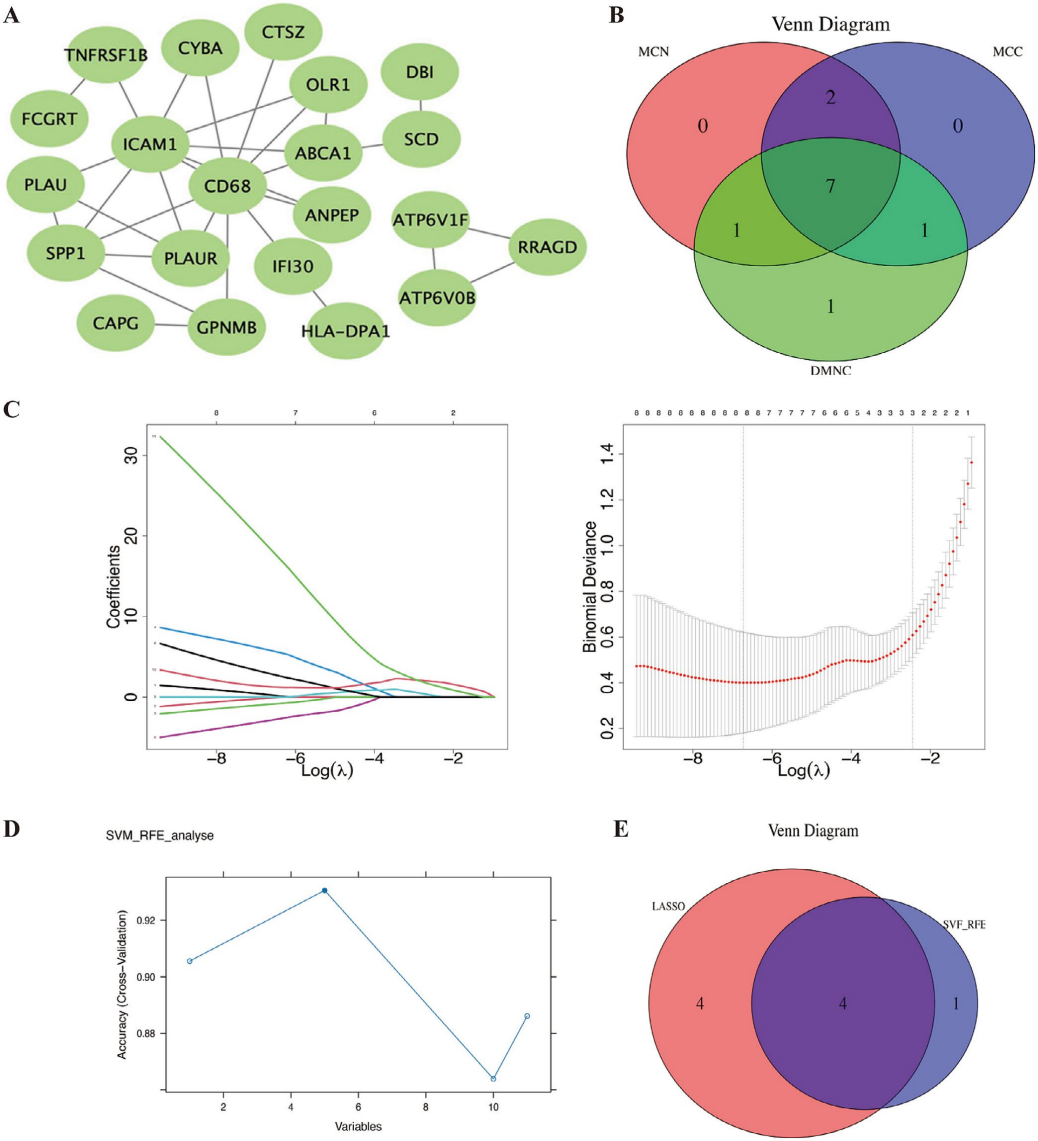
Identification of Hub genes. (A) Visualization of protein–protein interaction (PPI) network of the overlapping differentially expressed genes. (B) Use of three cytoHubba algorithms in Cytoscape software to identify hub genes in PPI network. (C) LASSO coefficient profile plot showing the selection of the optimal *λ* parameter. (D) The SVM‐RFE algorithm identified five crosstalk genes. (E) Venn diagram of genes screened by LASSO and SVM‐RFE.

To investigate the functional interactions among co‐expressed DEGs between VSMC‐derived foam cells and IPH, we constructed a PPI network using STRING (Figure 1A). The network comprised 40 nodes and 31 edges, representing key molecular interactions. To identify high‐confidence hub genes, we employed three distinct algorithms from the CytoHubba plugin in Cytoscape and extracted the top 10 ODEGs from each method. Consolidating these results yielded 30 candidate genes, of which 8 were consistently prioritized across multiple algorithms (Figure 1B).

**FIGURE 2:**
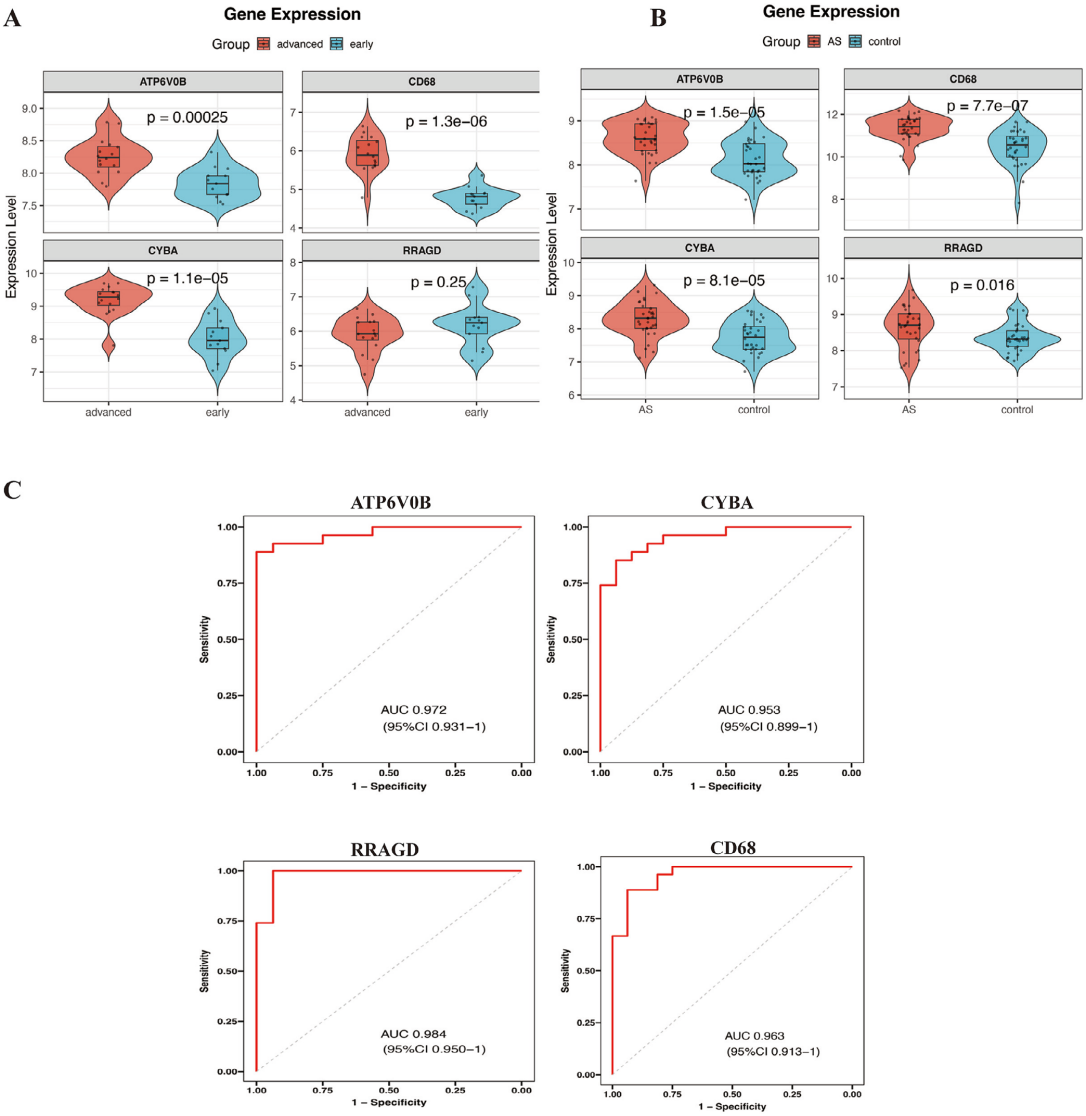
Validation of the four candidate hub genes. (A, B) Relative expression levels of the four candidate hub genes in datasets GSE28829 (A) and GSE43292 (B). (C) Receiver operating characteristic curve for four candidate hub genes in GSE163154.

To further refine the hub gene selection, we applied two machine learning approaches: LASSO regression and SVM‐RFE. LASSO regression identified eight candidate genes exhibiting the lowest binomial deviance (Figure 1C), while SVM‐RFE analysis revealed five genes with optimal CV accuracy and minimal CV error (Figure 2D). Intersection analysis between these two methods pinpointed four high‐confidence hub genes: CD68, CYBA, ATPase H+ Transporting V0 Subunit B (ATP6V0B), and Ras Related GTP Binding D (RRAGD) (Figure 1E).

### 3.5 Diagnostic Significance of Hub Genes

To validate the expression patterns of candidate hub genes in unstable atherosclerotic plaques, we analyzed two independent microarray datasets: GSE28829 (advanced atherosclerotic plaque vs. early atherosclerotic plaque) and GSE43292 (atheroma plaque vs. distant macroscopically intact tissue). In the GSE28829 dataset, CD68, CYBA, and ATP6V0B demonstrated substantial up‐regulation in advanced plaques, whereas the expression of RRAGD has no statistical significance (Figure 2A). In GSE43292, CD68, CYBA, ATP6V0B, and RRAGD were all upregulated in atheroma plaques (Figure 2B). Then, we constructed ROC curves for these candidates in the GSE163154 to calculate the AUC. The AUC values in all hub genes were higher than 0.9 (Figure 2C), suggesting their potential as biomarkers for plaque instability.

### 3.6 Immune Infiltration Analyses

**FIGURE 3:**
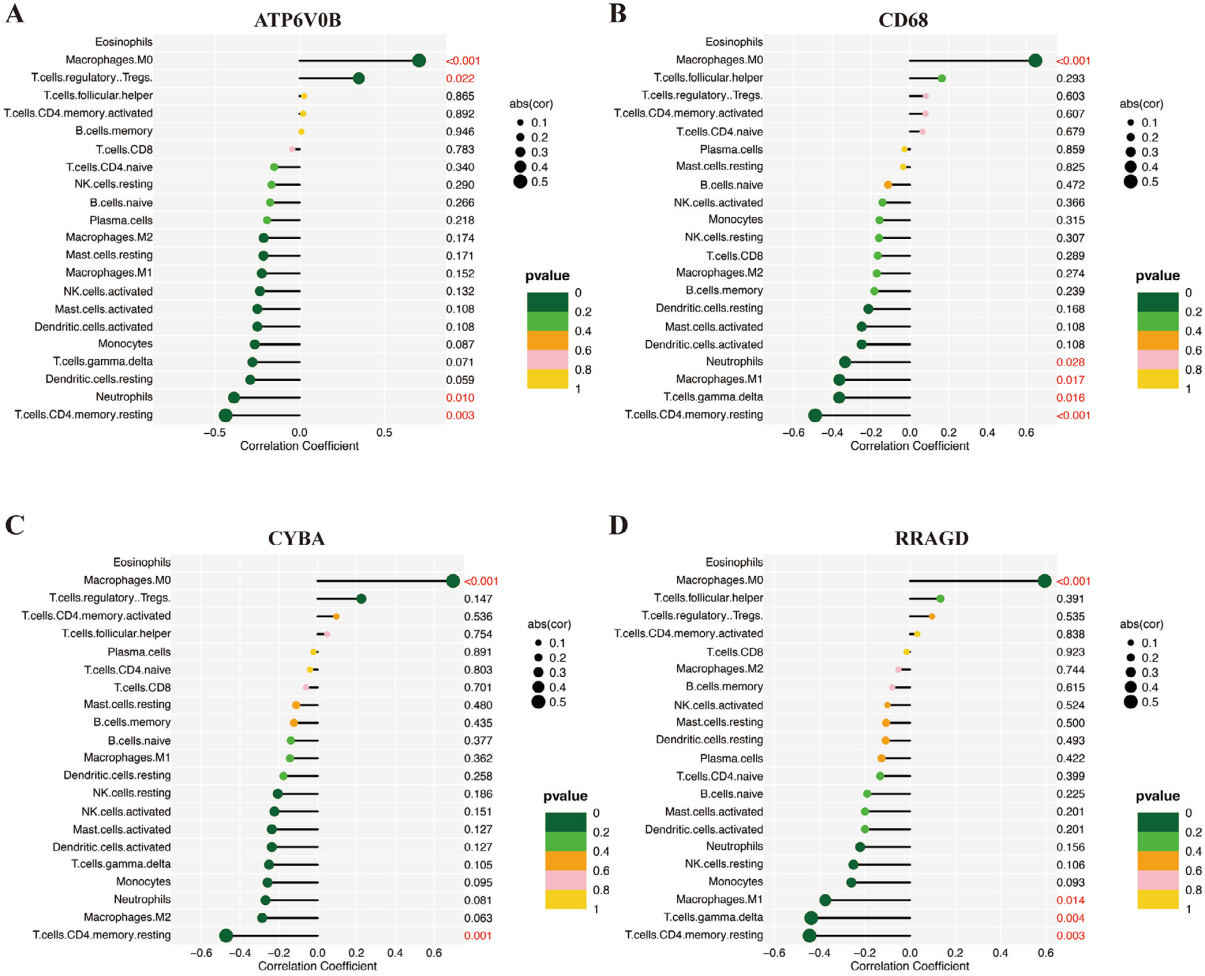
Immune infiltration analysis of the four hub genes. The correlation between the relative expression of (A) ATP6V0B, (B) CD68, (C) CYBA, and (D) RRAGD and the composition of immune cells in unstable plaques.

Immunocyte infiltration analysis of the GSE163154 dataset revealed that macrophage M0 infiltration was significantly reduced in IPH lesions compared to non‐IPH controls. In contrast, the proportion of CD4^+^ T memory resting cells and γδ T cells was significantly elevated in IPH samples (Figure S5A,B). Spearman rank correlation analysis demonstrated strong associations between these immune cell subsets and the hub genes (CD68, CYBA, ATP6V0B, and RRAGD) (Figure 3).

### 3.7 Experimental Validations of Hub Genes Expression in VSMC‐Derived Foam Cells

**FIGURE 4:**
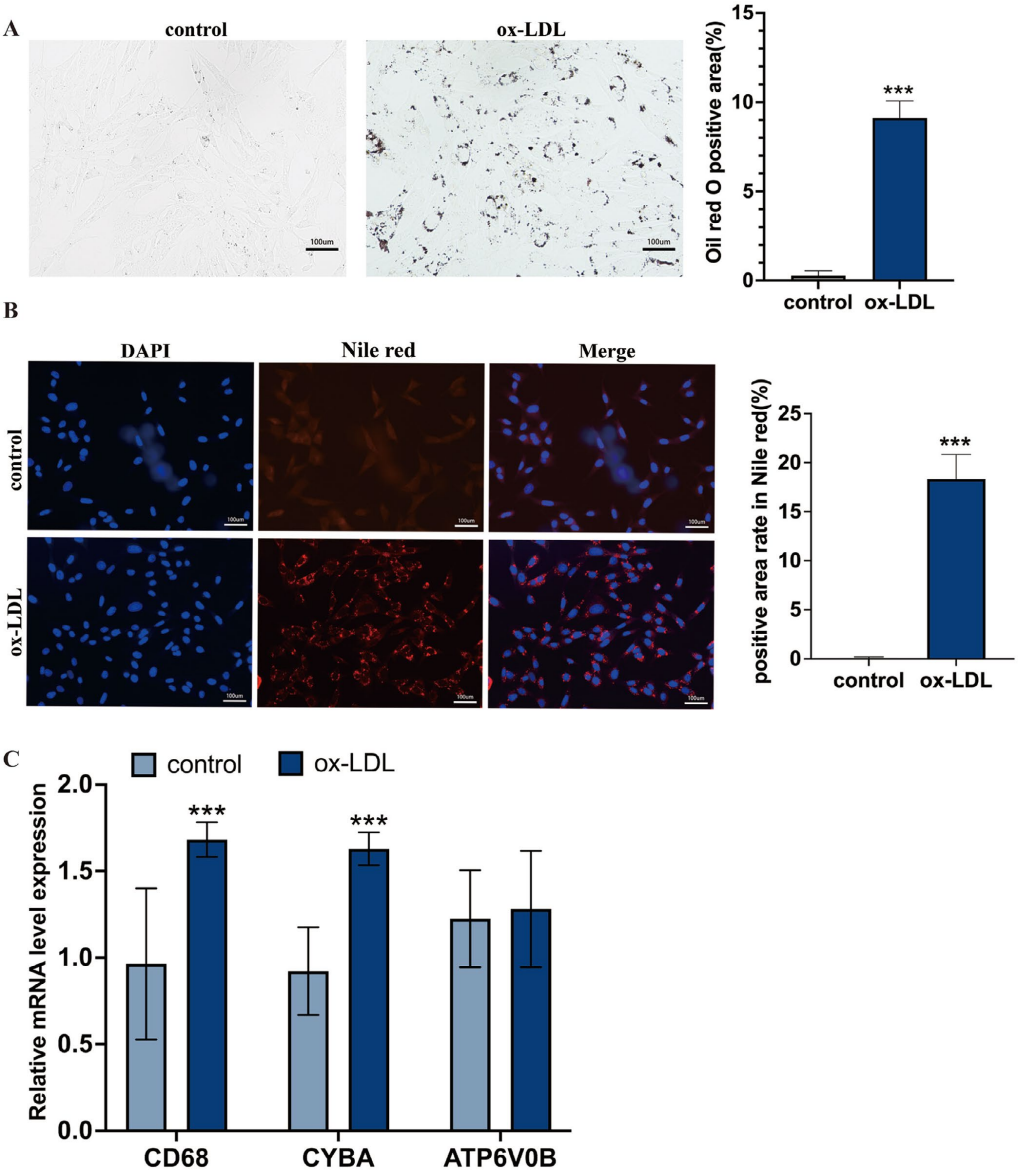
In vitro model of vascular smooth muscle cell (VSMC)‐derived foam cell formation. (A) Oil red O staining of VSMCs treated with or without oxidized low‐density lipoprotein (ox‐LDL). (B) Nile red staining of VSMCs treated with or without ox‐LDL; scale bars: 400 μm. (C) Measurement of mRNA expression levels of ATP6V0B, CD68 and CYBA. All data are expressed as mean ± standard deviation from three independent experiments; *p < 0.05, **p < 0.01, **p < 0.01 vs. without ox‐LDL.

ORO staining and Nile red fluorescence microscopy demonstrated that treatment of MOVAS with ox‐LDL for 24 h significantly increased lipid accumulation and foam cell production compared to untreated controls (Figure 4A,B). Consistent with phenotypic switching, the levels of VSMC contractile markers α‐SMA and SM22α were significantly downregulated following ox‐LDL treatment (Figure S6A). The mRNA expression levels of CD68, CYBA, and ATP6V0B in the VSMC‐derived foam cell model were assessed using RT‐qPCR. The results showed that CD68 and CYBA were significantly upregulated in ox‐LDL‐treated cells, whereas ATP6V0B expression did not reach statistical significance (Figure 4C). Based on these results, CD68 and CYBA were selected for further mechanistic studies, whereas ATP6V0B and RRAGD were excluded due to a lack of consistent validation in independent datasets (GSE28829) or the in vitro foam cell model, respectively.

### 3.8 The Hub Genes Expression in the Aortic Tissues of Atherosclerotic Mice

**FIGURE 5:**
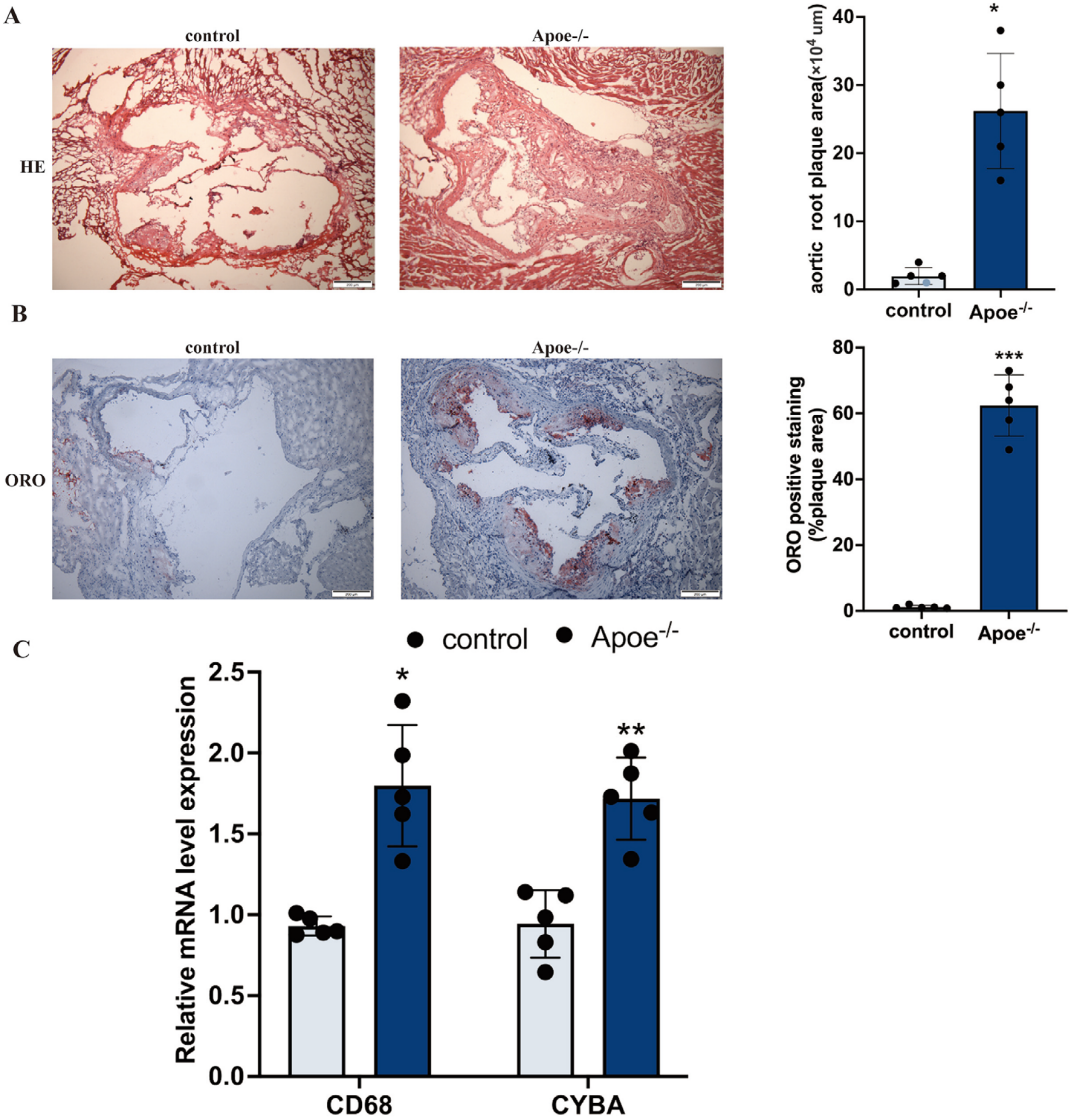
Confirmation of hub genes expression in atherosclerosis mice. (A) Hematoxylin–eosin staining of aortic root cross‐sections from ApoE^−/−^ and C57/BL6 mice (*n* = 5/group) with the plaque area quantification; scale bars: 400 μm. (B) Representative oil red O staining images of aortic root cross‐sections with the lesion volume quantification; scale bars: 400 μm. (C) The mRNA levels of aortic vascular tissues (*n* = 5/group). All data are expressed as mean ± standard deviation from three independent experiments; *p < 0.05, **p < 0.01 vs. C57/BL6 mice group.

We investigated the expression of CD68 and CYBA in an aortic atherosclerotic mouse model fed a HFD for 12 weeks. Consistent with the in vitro findings, H&E and ORO staining of the aortic root revealed prominent plaque formation in ApoE^−^/^−^ mice (Figure 5A,B). RT‐qPCR analysis demonstrated that the expression levels of CD68 and CYBA were significantly upregulated in the aortic tissues of atherosclerotic mice compared to the controls (Figure 5C).

### 3.9 FOXC1 as a Key TF of Hub Genes

**FIGURE 6:**
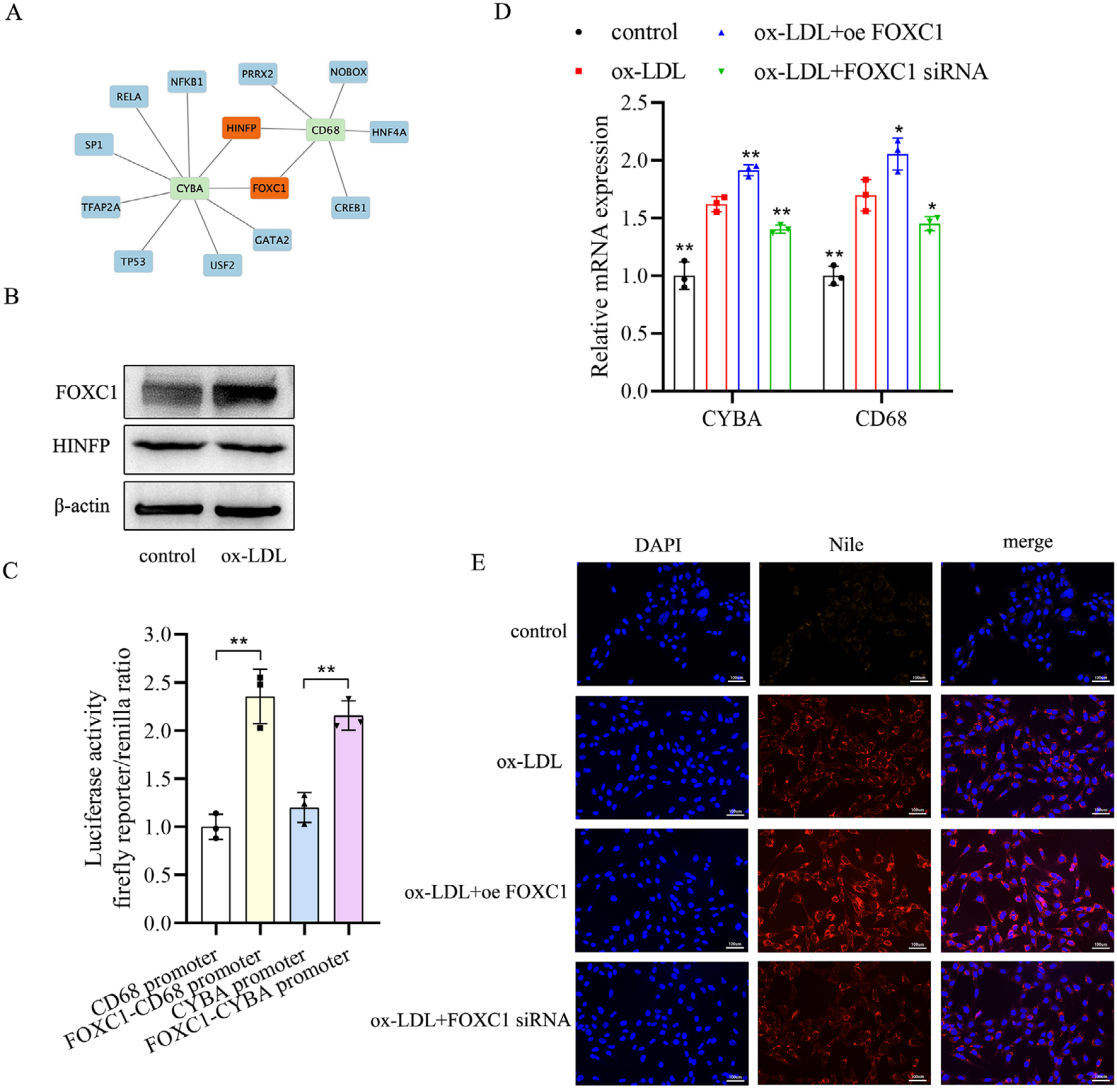
Identification of the upstream transcription factors of the hub gene. (A) Prediction of transcription factors regulating CD68 and CYBA using NetworkAnalyst online website. (B) FOXC1 and HINFP protein levels were examined in mouse aortic vascular smooth muscle cells (MOVAS) after ox‐LDL stimulation. (C) The effect of FOXC1 on the promoter activity of CD68 and CYBA in 293 T cells was detected by Dual‐luciferase reporter assay. (D) Analysis of CD68 and CYBA mRNA expression in MOVAS with FOXC1 overexpression or knockdown. (E) Nile red staining of MOVAS with FOXC1 overexpression or knockdown; scale bars: 400 μm. All data are expressed as mean ± standard deviation from three independent experiments; *p < 0.05, **p < 0.01 vs. ox‐LDL.

To explore the upstream regulatory mechanisms of the key genes, we predicted 9 TFs targeting the CYBA gene and 6 TFs targeting the CD68 gene, among which FOXC1 and HINFP were identified as common TFs (Figure 6A). In the MOVAS model stimulated by ox‐LDL, the FOXC1 protein significantly increased, while the HINFP protein showed no significant change (Figure 6B). Luciferase reporter assays showed that FOXC1 significantly activated the promoter activities of CD68 and CYBA in 293 T cells and MOVAS, indicating that CD68 and CYBA are target genes of FOXC1 (Figure 6C; Figure S6B). Consistently, overexpression of FOXC1 in MOVAS significantly upregulated the expression of CD68 and CYBA and enhanced lipid accumulation, whereas knockdown of FOXC1 exerted the opposite effects (Figure 6D,E; Figure S6C). Given that CYBA encodes a critical subunit of NADPH oxidase, we next examined whether FOXC1 modulates intracellular ROS levels in ox‐LDL‐treated MOVAS. FOXC1 overexpression significantly increased ROS production, whereas FOXC1 knockdown markedly reduced ROS levels, consistent with its regulation of CYBA expression (Figure S6D). Collectively, these findings demonstrate that FOXC1 functions as a crucial TF of CD68 and CYBA, promoting VSMC‐derived foam cell formation.

## 4 Discussion

AS is a chronic inflammatory disease characterized by lipid deposition and plaque formation in the arterial wall, serving as the pathological basis for numerous cardiovascular and cerebrovascular events [13]. As plaques progress, they enlarge and become unstable, ultimately rupturing and precipitating life‐threatening acute coronary syndromes [14, 15]. IPH represents a critical feature of plaque instability, associated with enhanced lipid deposition, macrophage recruitment, and necrotic core expansion, thereby exacerbating plaque vulnerability [16, 17, 18]. Lipid deposition can promote the formation of foam cells and the development of plaque [19]. Recent studies have found that 50% of foam cells in AS plaque are transdifferentiated from VSMCs [8, 20]. However, little is known about whether plaque hemorrhage affects the formation of foam cells.

In this study, we applied integrated bioinformatics approaches to identify key genes involved in VSMC‐derived foam cell formation and unstable plaque development. Hub genes were selected by intersecting DEGs with WGCNA‐derived IPH module genes. By constructing a protein–protein interaction network and integrating 3 algorithms from cytoHubba, LASSO and SVM‐RFE, we identified two hub genes (CD68 and CYBA) that exhibit high diagnostic accuracy in distinguishing atherosclerotic samples from controls. We have verified through other datasets of atherosclerotic plaques and in vitro and in vivo experiments that these genes are significantly upregulated during the transdifferentiation of VSMCs into foam cells. Notably, FOXC1 was identified as a common upstream TF significantly upregulated in VSMC‐derived foam cells. Dual‐luciferase assays confirmed that FOXC1 directly activates CD68 and CYBA promoters, and functional studies demonstrated that FOXC1 overexpression promotes CD68 and CYBA expression and lipid accumulation in VSMCs, while FOXC1 knockdown exerts opposite effects. These findings establish FOXC1 as a key transcriptional regulator of VSMC‐derived foam cell formation.

CD68 is a well‐established macrophage marker. Studies have demonstrated that when VSMCs undergo phenotypic transition into foam cells, their expression of smooth muscle cell markers decreases, while CD68 expression is upregulated [21]. Furthermore, utilizing multicolor‐labeling techniques and random recombinant fluorescent transgenic mouse models, accumulating evidence has revealed that atherosclerotic plaques contain macrophage‐like cells positive for CD68, galectin‐3, and lysosomal‐associated membrane protein 2. These cells originate from a distinct VSMC subpopulation [22, 23]. Therefore, CD68 can be used as a marker for VSMC to transform into foam cells. Consistent with these findings, we also found that the expression of CD68 in VSMC‐derived foam cells increased through screening and validation of bioinformatics analysis.

CYBA also known as p22phox, is a critical cytosolic subunit necessary for the activation of nicotinamide adenine dinucleotide phosphate (NADPH) oxidase [24]. The NADPH oxidase (NOX) complex serves as a primary source of reactive oxygen species (ROS) [25]. Notably, CYBA plays a pivotal role in modulating NOX‐dependent ROS production. Studies have shown that elevated CYBA expression and activity are associated with oxidative stress and inflammatory pathways [26, 27, 28]. Oxidative stress plays an important role in the proliferation, migration, and phenotype transformation of VSMCs [29]. Therefore, CYBA may promote the formation of VSMC derived foam cells by regulating the production of ROS in oxidative stress. Numerous studies have also confirmed that the CYBA polymorphism correlated with atherosclerosis [30]. These studies suggest a potential protective role of CYBA against atherosclerosis. Although both CYBA and atherosclerosis are linked to ROS, the role of CYBA in atherosclerosis remains unknown. This highlights the need for further research to uncover the intricate relationship between CYBA and atherosclerosis.

Atherosclerosis pathogenesis is closely associated with immune‐mediated inflammation, where metabolic alterations and immune responses are intricately interconnected [31, 32]. Utilizing CIBERSORT for global immune infiltration analysis of unstable plaques, we observed elevated proportions of macrophages, resting memory CD4+ T cells, and gamma delta T cells compared to stable plaques. Subsequent Spearman rank correlation analysis revealed significant associations between hub genes and immune cell infiltration. Notably, CD68 and CYBA expression exhibited strong correlations with macrophage abundance and T cell infiltration. Activated macrophages and foam cell formation serve as key drivers of unstable plaques, sustaining inflammatory responses, promoting plaque destabilization, and triggering thrombotic events [31, 33]. Among immune cell subsets, CD4+ T cells emerge as critically involved in all stages of atherosclerosis from initiation and progression to plaque regression, rupture, or erosion [34]. These studies indicate that immune cells play an important role in the formation of atherosclerotic plaque. Pro‐inflammatory T cells secrete pro‐inflammatory cytokines (such as IFN‐γ, TNF‐α, IL‐17), activate macrophages and endothelial cells, increase plaque vulnerability, and induce IPH. After plaque bleeding, red blood cells release hemoglobin, which can activate macrophages through Toll like receptor 4, release cytokines such as IL‐12 and IL‐23, and promote T cell polarization [17, 35, 36, 37]. However, more research is still needed to clarify the expression of these key genes in immune cells, and further investigation is also needed to understand the mechanisms by which these key genes affect immune cells.

A limitation of this study is the lack of human protein‐level validation, as we could not confirm FOXC1 expression in patients with intraplaque hemorrhage by IHC due to difficulty in obtaining human carotid plaque specimens. Additionally, the ApoE^−^/^−^ mouse model does not fully mimic spontaneous plaque hemorrhage as seen in humans. Future studies with human samples and more clinically relevant animal models are warranted to validate our findings.

## 5 Conclusions

In conclusion, this study identifies CD68 and CYBA as key hub genes in VSMC‐derived foam cell formation and unstable atherosclerotic plaques. We demonstrate for the first time that FOXC1 functions as a critical transcription factor regulating CD68 and CYBA expression, thereby promoting VSMC transdifferentiation into foam cells. These findings provide new insights into the molecular mechanisms of plaque instability and highlight FOXC1, CD68, and CYBA as potential diagnostic biomarkers and therapeutic targets for atherosclerosis.

## Funding

This work was supported by Basic Scientific Research Funds of Department of Education of Zhejiang Province (KYYB2023014), Zhejiang Provincial Health Industry Science and Technology Program (2025KY589), the National Natural Science Foundation of China [Grant Nos. 82100502] and the Zhejiang Provincial Basic Public Welfare Research Projects [Grant Nos. LQ22H020007].

## Conflicts of Interest

The authors declare no conflicts of interest.

## Supporting information


**Table S1:** Primer sequences used in RT‐qPCR.
**Figure S1:** A schematic view of the study's procedure.
**Figure S2:** Weighted gene co‐expression network analysis (WGCNA) identified gene modules significantly associated with unstable atherosclerotic plaques. (A) Scale‐free fitting indices and mean connectivity were used to determine the optimal soft‐threshold power in WGCNA for unstable plaques. (B) Clustering of sample. (C) Clustering dendrogram, where different colors denote different gene modules. (D) Heatmap depicting the relationships between module characteristic genes and clinical features. (E) Scatterplot of genes in the blue module.
**Figure S3:** Volcano plots and heatmaps illustrating differentially expressed genes (DEGs) in datasets GSE163154 and GSE68021. (A, C) Volcano plots of all DEGs in GSE163154 and GSE68021, respectively. Up (red region): expression upregulation; down (blue region): expression downregulation; Non (Gray region): no statistical difference. (B, D) Heatmaps of the top 50 statistically significant genes in GSE163154 and GSE68021, respectively.
**Figure S4:** Identification of overlapping differentially expressed genes (ODEGs) between human vascular smooth muscle cell‐derived foam cells and human carotid atheroma intraplaque hemorrhage tissues, followed by functional enrichment analysis. (A) Venn diagram of differentially expressed genes (DEGs) from datasets GSE163154 and GSE68021 with genes in the blue module identified via weighted gene co‐expression network analysis (WGCNA) of GSE163154. (B) Gene Ontology (GO) enrichment analysis results for the ODEGs identified in the Venn diagram. (C) Kyoto Encyclopedia of Genes and Genomes (KEGG) pathway enrichment analysis of the ODEGs from the Venn diagram.
**Figure S5:** Immune cell infiltration analysis. (A) Stacked bar chart displaying the composition of immune cells. (B) Comparison of immune cell proportions between the intraplaque hemorrhage (IPH) and non‐IPH groups. *p < 0.05.
**Figure S6:** Validation of FOXC1 target genes and functional effects in mouse aortic vascular smooth muscle cells (MOVAS). (A) α‐SMA and SM22α protein levels in MOVAS after ox‐LDL stimulation. (B) Dual‐luciferase reporter assay of CD68 and CYBA promoter activities in MOVAS with FOXC1 overexpression. (C) CD68 and CYBA protein levels in MOVAS with FOXC1 overexpression or knockdown. (D) Intracellular reactive oxygen species (ROS) levels in MOVAS with FOXC1 overexpression or knockdown under ox‐LDL stimulation. All data are expressed as mean ± standard deviation from three independent experiments; *p < 0.05, **p < 0.01, ***p < 0.001 vs. ox‐LDL.

## Data Availability

The data that support the findings of this study are available from the corresponding author upon reasonable request.
