# Supplementary material for: FOXC1: A Key Transcription Factor of VSMC‐Derived Foam Cell Formation in Atherosclerotic Plaque Instability
Source: Kaohsiung J Med Sci. 2026 Jul 20:e70269. Online ahead of print. doi: 10.1002/kjm2.70269 (PMC13399657; doi:10.1002/kjm2.70269)
Supplement: Supplementary file 1 — Table S1: Primer sequences used in RT‐qPCR. Figure S1: A schematic view of the study's procedure. Figure S2: Weighted gene co‐expression network analysis (WGCNA) identified gene modules significantly associated with unstable atherosclerotic plaques. (A) Scale‐free fitting indices and mean connectivity were used to determine the optimal soft‐threshold power in WGCNA for unstable plaques. (B) Clustering of sample. (C) Clustering dendrogram, where different colors denote different gene modules. (D) Heatmap depicting the relationships between module characteristic genes and clinical features. (E) Scatterplot of genes in the blue module. Figure S3: Volcano plots and heatmaps illustrating differentially expressed genes (DEGs) in datasets GSE163154 and GSE68021. (A, C) Volcano plots of all DEGs in GSE163154 and GSE68021, respectively. Up (red region): expression upregulation; down (blue region): expression downregulation; Non (Gray region): no statistical difference. (B, D) Heatmaps of the top 50 statistically significant genes in GSE163154 and GSE68021, respectively. Figure S4: Identification of overlapping differentially expressed genes (ODEGs) between human vascular smooth muscle cell‐derived foam cells and human carotid atheroma intraplaque hemorrhage tissues, followed by functional enrichment analysis. (A) Venn diagram of differentially expressed genes (DEGs) from datasets GSE163154 and GSE68021 with genes in the blue module identified via weighted gene co‐expression network analysis (WGCNA) of GSE163154. (B) Gene Ontology (GO) enrichment analysis results for the ODEGs identified in the Venn diagram. (C) Kyoto Encyclopedia of Genes and Genomes (KEGG) pathway enrichment analysis of the ODEGs from the Venn diagram. Figure S5: Immune cell infiltration analysis. (A) Stacked bar chart displaying the composition of immune cells. (B) Comparison of immune cell proportions between the intraplaque hemorrhage (IPH) and non‐IPH groups. *p < 0.05. Figure S6: Validation of FOXC1 ta [file KJM2-9999-e70269-s001.docx]

**Supplementary Table 1** Primer sequences used in RT-qPCR.

| **Gene** | **Forward (5′–3′)** | **Reverse (5′–3′)** |
| --- | --- | --- |
| ATP6VOB | AGACTGCGGGACGGACGG | TGACCCAGACAAATACCAAGT |
| CYBA | ATTTCACAAGGCGCCGAAAC | AATGCACAGGAGGGTGACTG |
| CD68 | AGCCACAAAACCACCACTCA | CTAGTGGTGGCAGGACTGTG |
| β-actin | CTACCTCATGAAGATCCTGACC | CACAGCTTCTCTTTGATGTCAC |

**
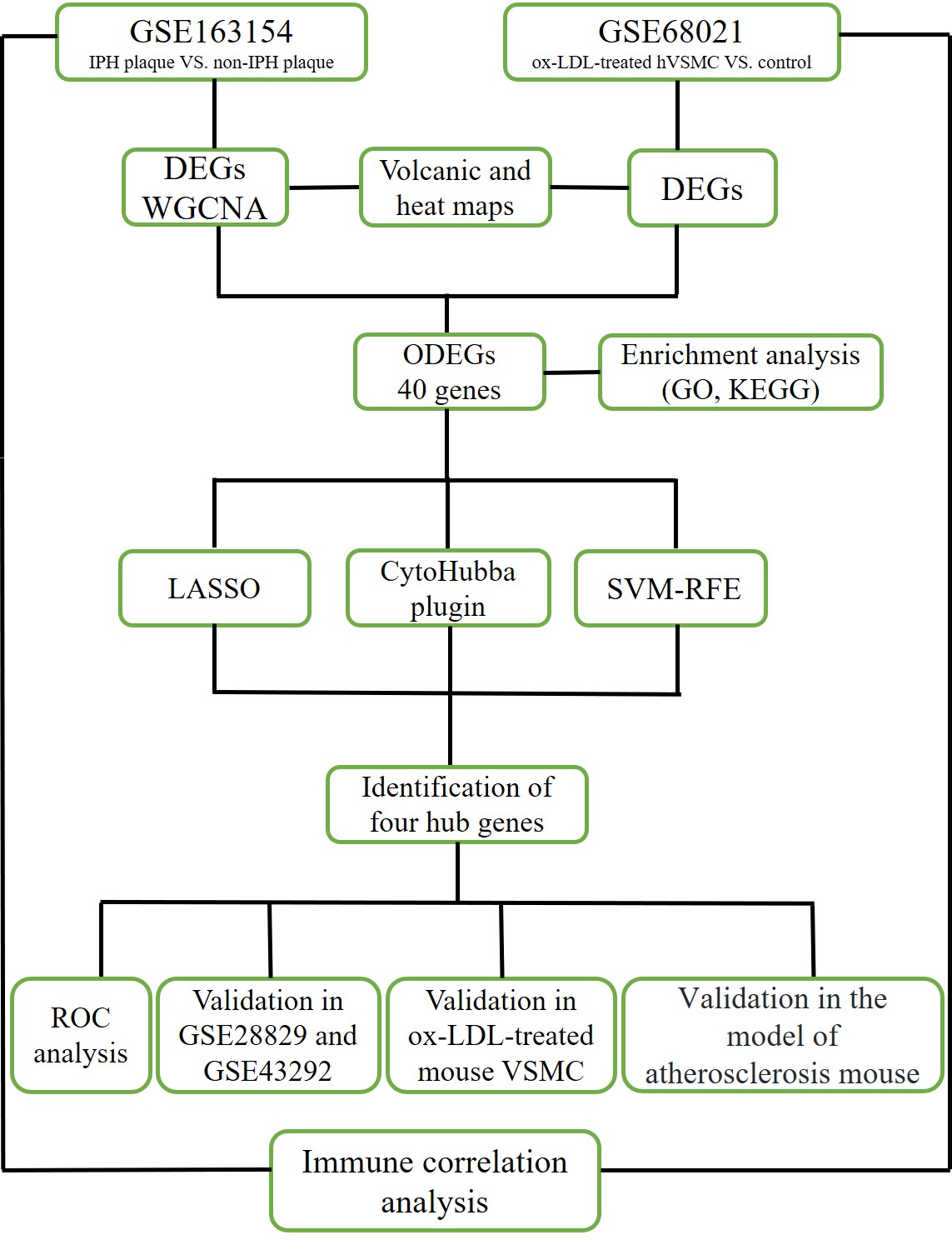
** **Supplementary Figure 1** A schematic view of the study’ s procedure.


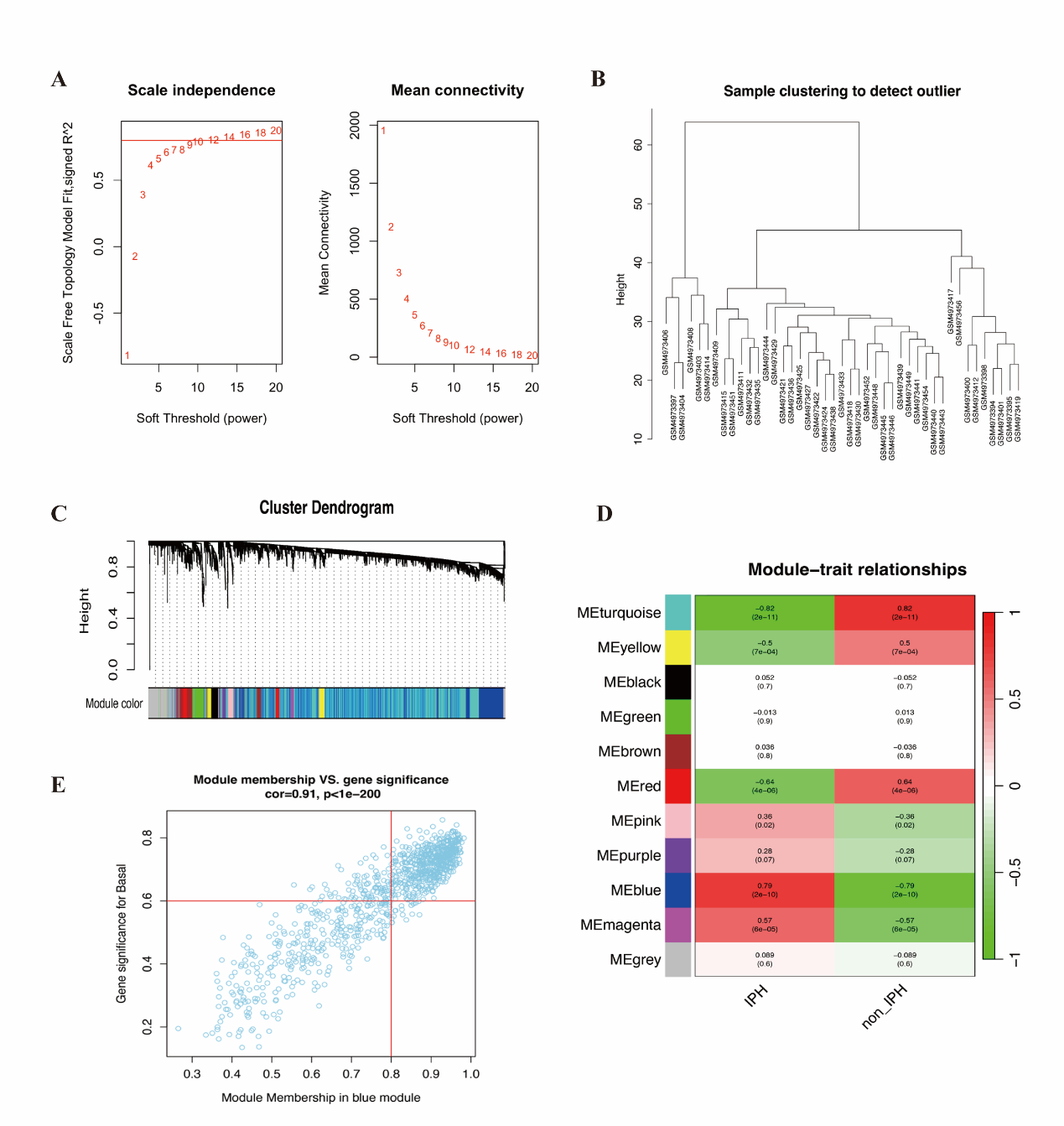


**Supplementary Figure 2 Weighted gene co-expression network analysis (WGCNA) identified gene modules significantly associated with unstable atherosclerotic plaques.** (A) Scale-free fitting indices and mean connectivity were used to determine the optimal soft-threshold power in WGCNA for unstable plaques. (B) Clustering of sample. (C) Clustering dendrogram, where different colors denote different gene modules. (D) Heatmap depicting the relationships between module characteristic genes and clinical features. (E) Scatterplot of genes in the blue module.

**
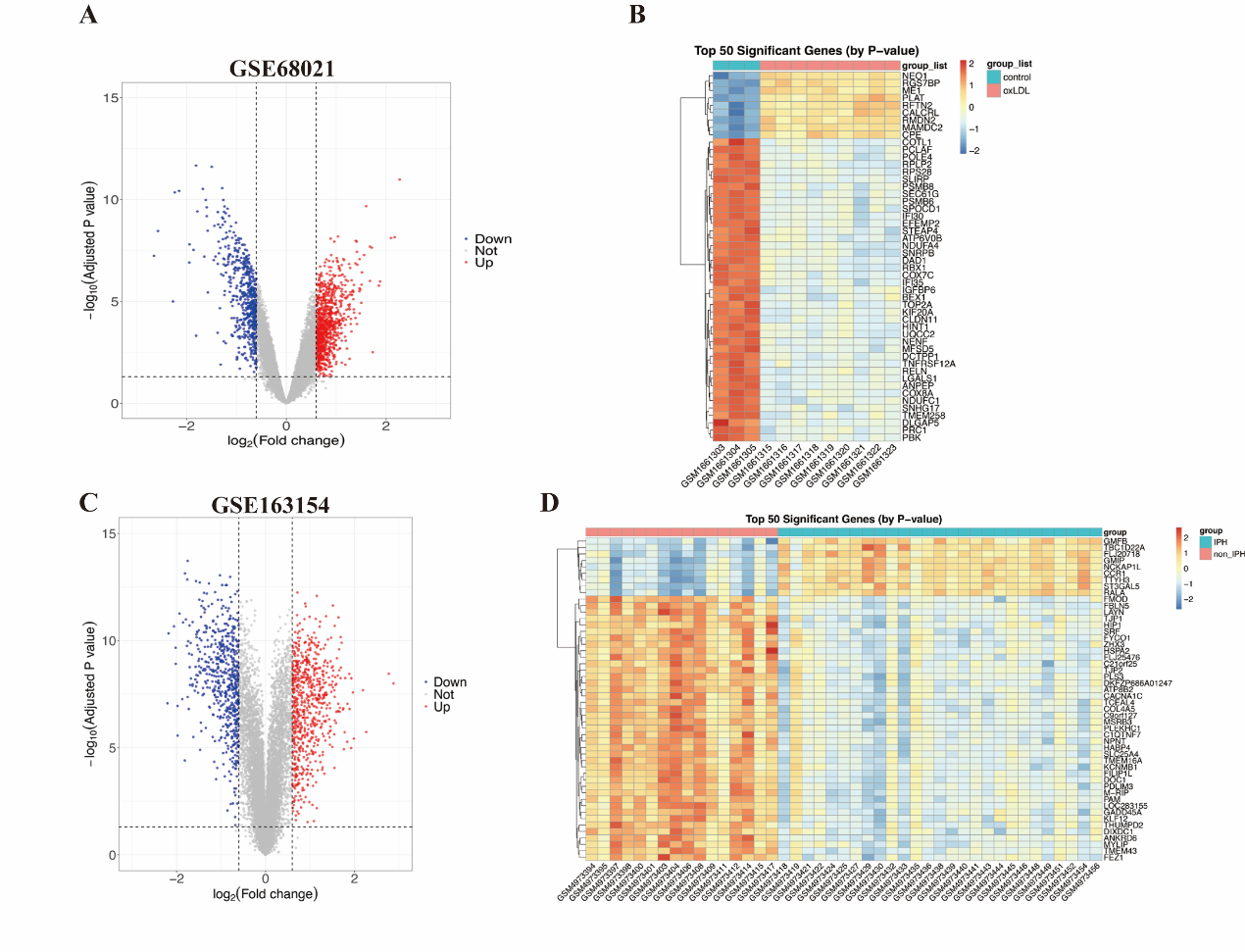
**

**Supplementary Figure 3** Volcano plots and heatmaps illustrating differentially expressed genes (DEGs) in datasets GSE163154 and GSE68021. (**A**) and (**C**) Volcano plots of all DEGs in GSE163154 and GSE68021, respectively. Up (red region): expression upregulation; down (blue region): expression downregulation; Non (Gray region): no statistical difference. (**B**) and (**D**) Heatmaps of the top 50 statistically significant genes in GSE163154 and GSE68021, respectively.


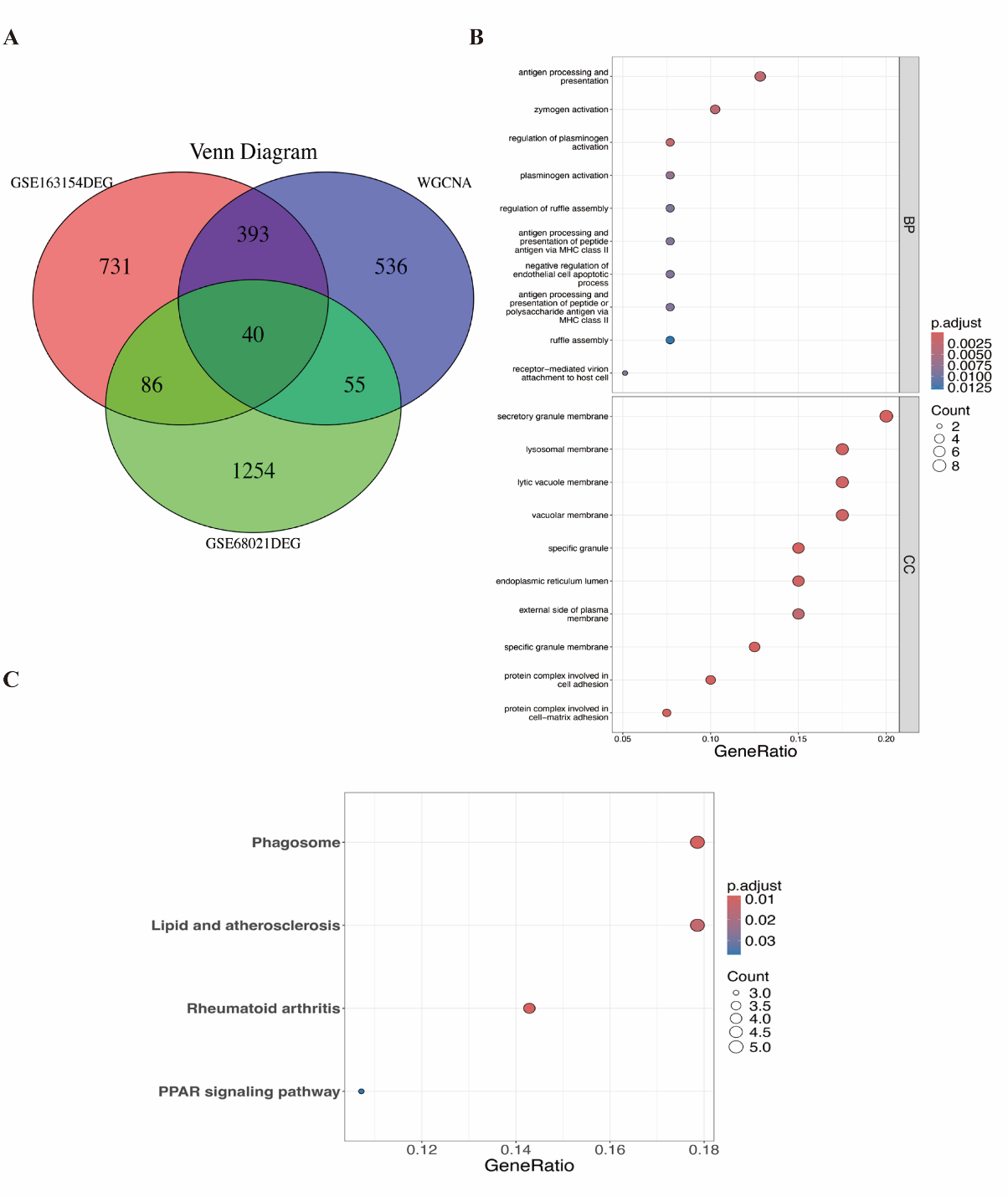


**Supplementary Figure 4** Identification of overlapping differentially expressed genes (ODEGs) between human vascular smooth muscle cell-derived foam cells and human carotid atheroma intraplaque hemorrhage tissues, followed by functional enrichment analysis. (**A**) Venn diagram of differentially expressed genes (DEGs) from datasets GSE163154 and GSE68021 with genes in the blue module identified via weighted gene co-expression network analysis (WGCNA) of GSE163154. (**B**) Gene Ontology (GO) enrichment analysis results for the ODEGs identified in the Venn diagram. (**C**) Kyoto Encyclopedia of Genes and Genomes (KEGG) pathway enrichment analysis of the ODEGs from the Venn diagram.


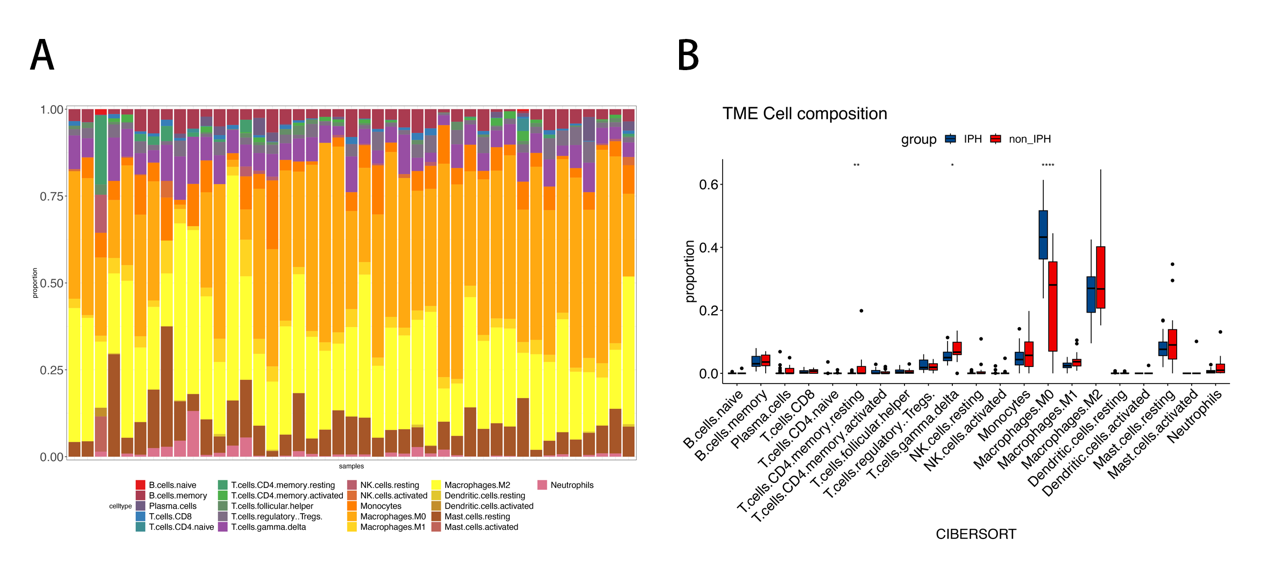


**Supplementary Figure 5** Immune cell infiltration analysis. (**A**) Stacked bar chart displaying the composition of immune cells. (**B**) Comparison of immune cell proportions between the intraplaque hemorrhage (IPH) and non-IPH groups. **p* < 0.05.

**
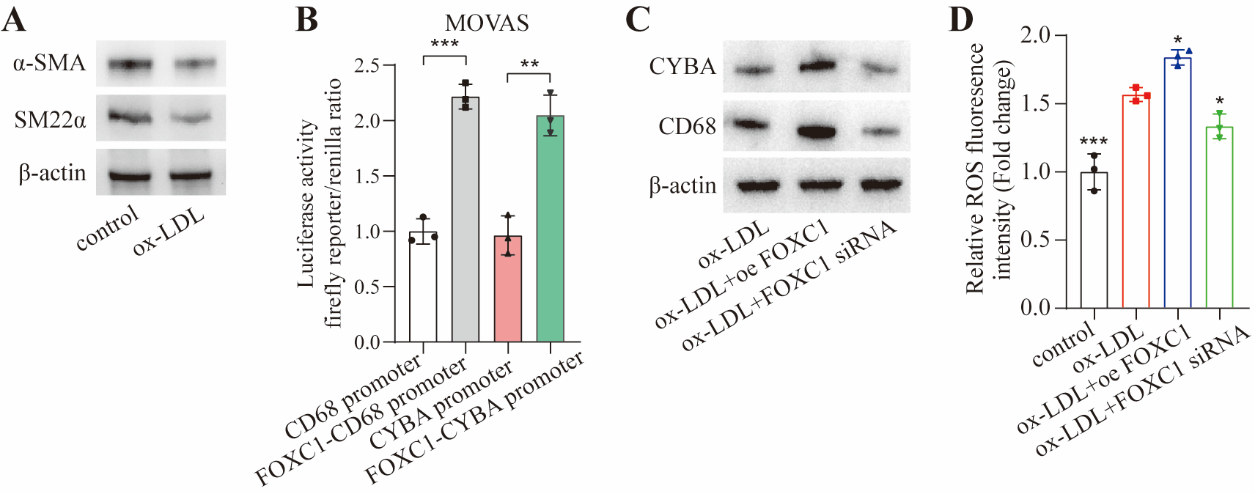
**

**Supplementary Figure 6** Validation of FOXC1 target genes and functional effects in mouse aortic vascular smooth muscle cells (MOVAS). (**A**) α-SMA and SM22α protein levels in MOVAS after ox-LDL stimulation. (**B**) Dual-luciferase reporter assay of CD68 and CYBA promoter activities in MOVAS with FOXC1 overexpression. (**C**) CD68 and CYBA protein levels in MOVAS with FOXC1 overexpression or knockdown. (**D**) Intracellular reactive oxygen species (ROS) levels in MOVAS with FOXC1 overexpression or knockdown under ox-LDL stimulation. All data are expressed as mean ± standard deviation from three independent experiments; **p*<0.05, ***p*<0.01, ****p*<0.001 vs. ox-LDL.
